# Co-twin design in brain imaging—review on biomarkers of Alzheimer's disease

**DOI:** 10.1093/cercor/bhad181

**Published:** 2023-05-25

**Authors:** Anni Varjonen, Claudia Schwarz, Eero Vuoksimaa

**Affiliations:** Institute for Molecular Medicine Finland (FIMM), HiLIFE, University of Helsinki, Helsinki 00014, Finland; Institute for Molecular Medicine Finland (FIMM), HiLIFE, University of Helsinki, Helsinki 00014, Finland; Department of Neurology, University Medicine Greifswald, Greifswald 17475, Germany; Institute for Molecular Medicine Finland (FIMM), HiLIFE, University of Helsinki, Helsinki 00014, Finland

**Keywords:** cognitive impairment, dementia, magnetic resonance imaging, positron emissions tomography, twins

## Abstract

Twin samples allow to conduct a quasi-experimental co-twin case–control approach that can control for genetic and environmental confounding in brain–cognition associations, being more informative on causality compared with studies in unrelated individuals. We conducted a review of studies that have utilized discordant co-twin design to investigate the associations of brain imaging markers of Alzheimer's disease and cognition. Inclusion criteria encompassed twin pairs discordant for cognition or Alzheimer's disease imaging markers and reporting of within-twin pair comparison on the association between cognition and brain measures. Our PubMed search (2022 April 23, updated 2023 March 9) resulted in 18 studies matching these criteria. Alzheimer's disease imaging markers have been addressed only by few studies, most with small sample size. Structural magnetic resonance imaging studies have indicated greater hippocampal volume and thicker cortex in co-twins with better cognitive performance compared with their co-twins with poorer cognitive performance. No studies have looked at cortical surface area. Positron emission tomography imaging studies have suggested that lower cortical glucose metabolism rate and higher cortical neuroinflammation, amyloid, and tau accumulations are related to poorer episodic memory in within-twin pair comparisons. Thus far, only cross-sectional within-twin pair associations of cortical amyloid and hippocampal volume with cognition have been replicated.

Twin studies have contributed much to our understanding of the importance of genetic effects in brain and cognitive development and aging, and indicated substantial genetic influences on brain–cognition associations (Kremen et al. 2023). Most twin studies using in vivo brain imaging have utilized classical ACE models and bi- and multivariate extensions such as Cholesky decomposition. In the ACE model, the relative proportions of additive genetic (A) as well as common (C) and unique (E) environmental effects can be estimated by comparing the similarity of monozygotic (MZ) and dizygotic (DZ) twin pairs without any measured genes. Despite the availability of DNA-based polygenic scores summarizing the small effects of thousands of single-nucleotide polymorphisms in unrelated individuals, twin studies are still a valuable resource as the polygenic scores for brain and cognitive phenotypes do not explain the whole genetic variance obtained from twin studies.

Furthermore, in addition to ACE models investigating genetic and environmental underpinnings of brain health, cognition, and their relationships, twin samples can be used to conduct co-twin and within-twin pair analyses. A quasi-experimental discordant co-twin approach (a special kind of matched case–control design) can inform more about causal mechanisms from brain imaging markers to cognition compared with studies in unrelated individuals (Panizzon et al. 2022). In a case–control co-twin design, it is possible to study either cognitively discordant twin pairs or twin pairs who are discordant for brain measures such as neurodegeneration or amyloid pathology. In within-twin pair analyses, it is possible to use continuous brain and cognitive measures to see if the between-family (individual-level) associations are evident in within-family comparisons. These models control for shared environmental (fully in DZ and MZ pairs) and genetic (partly in DZ and fully in MZ) influences. Although not being a definite test of causality, these co-twin comparisons are closest to experimental design in humans as it is not possible to randomly assign individuals to high versus low brain pathology or to impaired versus intact memory groups.

In this review, we focused on studies that have utilized discordant co-twin design to investigate brain–cognition associations in aging in relation to brain imaging markers of Alzheimer's disease (AD). We will first discuss AD-related brain characteristics and pathology and their associations with cognition, followed by an introduction to co-twin design and a review of co-twin studies using in vivo brain imaging measures of AD.

## Biomarkers of AD

AD is defined by its 2 pathological hallmarks, extracellularly amyloid-beta (amyloid-β) plaques and intracellularly neurofibrillary tangles (NFTs) of hyperphosphorylated tau, accompanied by progressive neurodegeneration especially in medial temporal lobe structures (Jack et al. 2018; DeTure and Dickson 2019). These underlying pathophysiological alterations initiate many years, even decades, before the diagnosis of AD (Bateman et al. 2012; Jack et al. 2013; Sperling et al. 2013; Masters et al. 2015). Moreover, these biomarkers are related to episodic memory impairment, which becomes apparent in preclinical stages of AD, such as mild cognitive impairment (MCI)—a heterogenous condition that confers a risk for AD.

More than a 100 years ago, AD was diagnosed mainly by postmortem analyses (autopsies) that focused on the presence of amyloid-β plaques and NFTs in the brains of individuals clinically diagnosed with dementia (Alzheimer 1911; Alzheimer et al. 1995). Today, AD diagnosis is based on clinical assessments and exclusion of other causes for cognitive decline. To increase diagnostic certainty especially in the early stages of AD, reliable and sensitive in vivo brain imaging biomarkers have been established to detect pathological changes in the continuum of AD (Henriques et al. 2018; Zetterberg and Schott 2019). In addition to the temporal detection of AD biomarkers, brain imaging measures also provide spatial information and allow to investigate specific brain regions in relation to cognition. In addition to brain imaging markers, also cerebrospinal fluid (CSF) samples are established as valid measures of amyloid and tau (Hampel et al. 2021), but these are not included in the scope of our review. There are also recent advances in blood-based biomarkers of AD (Hampel et al. 2021), but also these non-brain-imaging markers are out of the scope of our review.

Amyloid-β (A), tau (T), and neurodegeneration (N), form the biological research framework, called AT(N) framework, created by the National Institute on Aging and Alzheimer's Association (Jack et al. 2016, 2018). In this framework, the biological definition of AD is based on the biomarker status irrespectively of the cognitive status (Jack et al. 2016, 2018). This framework is open for novel biomarkers and indeed, extension of this model, namely ATX(N), has proposed potential novel (X) biomarkers such as Triggering Receptor Expressed on Myeloid Cells 2 (neuroinflammation), soluble platelet-derived growth factor receptor beta (microvascular pathology) and neurogranin (synaptic dysfunction; Hampel et al. 2021). Notably, around 10–30% of individuals clinically diagnosed with AD do not show AD-specific pathology. Reasons for the inconsistency between cognitive and biomarker status are still poorly understood. Thus, definition of AD as a biological construct, and improvement of our understanding of the association between cognition and biomarkers of AD, can help in early detection and in selecting individuals for interventions.

## Episodic memory impairment as a clinical hallmark of AD

Along the continuum of AD, initial cognitive changes are most apparent in episodic memory, with subtle deterioration measurable already in preclinical stages (Tromp et al. 2015; Schindler et al. 2017). Therefore, measuring episodic memory is a sensitive marker to differentiate AD from other dementia causing neurodegenerative diseases in early stages of the disease (Weintraub et al. 2012) and to assess the severity of AD (Schindler et al. 2017). First clinically relevant impairments in cognitive function that do not interfere with activities of daily living are seen in patients with MCI, who are at higher risk for developing AD (Gauthier et al. 2006; Petersen 2011). Previous studies have provided evidence for an association of episodic memory and AD biomarkers in unrelated individuals. Here, we discuss some findings based on imaging markers that are within the focus of our review.

### Positron emission tomography markers

Positron emission tomography (PET) measures in vivo molecular processes in the brain by using a wide range of radioactive tracers, making it a useful biomarker for identifying amyloid-β and pathological tau aggregations as well as neurodegeneration and neuroinflammation in the brain of the elderly (Chandra et al. 2019; Thientunyakit et al. 2022; Tripathi and Murray 2022). Cortical amyloid-β plaque deposition is commonly assessed with Pittsburgh Compound B (PiB) or fluorinated tracers and has been the focus of PET research for the last 25 years. The detection of hyperphosphorylated tau in NFTs using PET radiotracers has evolved greatly in recent years, in part because of the failure of anti-amyloid therapies in AD and increasing evidence that tau may precede amyloid and trigger the AD process (Yoshida et al. 2019). However, this approach for measuring tau is more challenging than amyloid-PET because of various issues such as a large number of nonspecific off-target binding sites and the ability to cross the blood–brain barrier neuronal cell membrane (Bischof et al. 2021; Yap et al. 2021; Thientunyakit et al. 2022). Following the first-generation tau PET tracers (e.g. ^18^F-flortaucipir), promising new PET ligands, so-called second-generation PET tracers, are currently being developed for clinical applications. Neurodegeneration or neuronal injury represents another category of AD biomarker, which is defined by the progressive neuronal loss associated with impaired neuronal function and can be measured by brain glucose metabolism using fluorodeoxyglucose (FDG) PET imaging (Thientunyakit et al. 2022; Tripathi and Murray 2022). In the AD continuum, glucose hypometabolism reflects neuronal dysfunction leading to a decrease in cerebral blood flow in specific brain regions and resulting in lower glucose delivery, which was observed already in preclinical AD stages. Neuroinflammation can be measured by PET using various tracers that mainly target the translocator protein 18 kDa, which is known to be upregulated in neuroinflammation (Chandra et al. 2019; Thientunyakit et al. 2022).

Amyloid-β aggregation has been shown to be associated with cognitive decline (e.g. in episodic memory performance) in older individuals (Jansen et al. 2018; Thientunyakit et al. 2022). However, there is some heterogeneity in studies that examined the association of cortical amyloid-β load and cognitive function, since comparable studies failed to show a direct relationship between global cortical amyloid-β load and memory performance (Mormino et al. 2012; Maass et al. 2018; Thientunyakit et al. 2022). A recent study also provided evidence that cognitive decline might precede pathological levels of amyloid-β and also predicts subsequent amyloid-β positivity (Elman et al. 2020).

The spread of NFTs in the early phase of the AD continuum has been shown to be associated with a decrease in memory performance in older individuals and might even predict episodic memory decline (Schöll et al. 2016; Maass et al. 2018; Knopman et al. 2019). Specifically, medial temporal lobe structures, including the hippocampus and entorhinal cortex, are known to be important for memory function and are affected by tau early in the course of AD (Braak and Braak 1995; Tripathi and Murray 2022). Furthermore, there is evidence that even in cognitively normal amyloid-β-negative individuals, poorer cognitive performance is associated with medial temporal and neocortical tau load (Villemagne et al. 2018; Thientunyakit et al. 2022). In addition, progressive accumulation of cortical tau in individuals with amyloid-β pathology has been associated with increasing cognitive impairment. Tau imaging is suggested to be more closely related to cognitive decline and neurodegeneration than amyloid-β imaging and, therefore, tau PET is a useful approach for accurate AD staging and its prognosis (Schwarz et al. 2018; Bucci et al. 2021).

Glucose metabolism in temporoparietal regions has shown to be associated with memory function and has the ability to predict future cognitive decline and conversion to AD (Thientunyakit et al. 2022; Tripathi and Murray 2022). Studies have also shown alterations of glucose metabolism and synaptic function in regions following the same pattern as for NFTs (Mosconi 2005; Strom et al. 2021). Moreover, previous PET studies have shown inconsistent findings on the association of neuroinflammation with cognitive impairment and episodic memory decline (Chandra et al. 2019).

### Magnetic resonance imaging markers

Another approach to measure neurodegeneration or neuronal injury is the use of noninvasive structural magnetic resonance imaging (MRI). Atrophy of the medial temporal lobe (e.g. hippocampus, entorhinal cortex) and other (sub)cortical gray matter regions has been shown to be associated with cognitive functions (e.g. memory). Moreover, MRI measures are proxies for neurodegeneration already in early stages of the AD continuum and for predicting further cognitive decline and disease progression in the elderly (O'Shea et al. 2016; Pelletier et al. 2017; Ten Kate et al. 2017; van Oostveen and de Lange 2021).

By definition, cortical volume is the product of cortical thickness (CTH) and cortical surface area (CSA), with both following different trajectories during aging (Vijayakumar et al. 2016). While CTH has been studied intensively, CSA is much less explored (Wierenga et al. 2014). Previous findings suggest that CTH is associated with disease severity and cognitive impairment (van Oostveen and de Lange 2021). Furthermore, studies have shown that CTH and CSA have different associations with cognition both at global and regional levels (Vuoksimaa et al. 2015, 2016). In addition, a recent study showed that imaging biomarkers including tau, amyloid, and CTH were strongest associated with memory function in the entorhinal cortex as well as, to a lesser extent, in other temporal regions in healthy older individuals (Knopman et al. 2019).

Taken together, the diagnosis of AD has evolved from the identification of amyloid-β plaques and NFTs in autopsies to sensitive brain imaging biomarkers that are valid in vivo proxies for neuropathological and cognitive changes in the AD continuum. However, the vast majority of research has been conducted in unrelated individuals, a design that does not allow to control for genetic and environmental confounding in brain–cognition associations. However, family studies, especially twin studies, can control for genetic confounders even without any measured genes and for all environmental factors that are shared between family members: compared with studies in unrelated individuals, studying within-family associations in twin pairs is more informative on the causality of brain–cognition associations.

## Co-twin design

Within-family analyses in full twin pairs can inform if brain–cognition associations are evident when controlling for shared genetic and environmental effects. There is no confounding if within-twin pair associations are significant and not different from individual-level association. If the brain–cognition association is evident in between-family (individual-level) analyses but not in within-family comparisons, then genetic and/or environmental confounding occurs. Whether the confounding is because of shared genetic or environmental effects—or both—can be further investigated by comparing the within-family associations separately in MZ and DZ twin pairs (Panizzon et al. 2022). If there is a significant association also in within-family comparisons, then there is also stronger evidence for the causality.

Although not a definite test of causality, co-twin design can inform more about causality of brain–cognition associations compared with studies in unrelated individuals. Within-twin pair comparisons are fully controlling for factors that are shared between co-twins including, for example, age, sex, and family factors, such as parental socio-economic status and parental education. These models control for all environmental factors that are shared between members of a twin pair (both in DZ and MZ). Furthermore, genetic effects are controlled, partly in DZ—who share on average 50% of their segregating genes—and fully in MZ—who are genetically identical. In other words, this quasi-experimental case–control design can test if brain–cognition associations are evident when controlling for genetic and environmental confounding. However, we note that within-twin pair estimates are not confounded by factors that are shared between co-twins, but the estimates can be biased in the case when co-twins do not share confounders or because of measurement error (see e.g. Frisell et al. 2012).

## Dichotomic outcome

When using dichotomic discordance, it is possible to cross-tabulate pairs with regard to discordance and concordance of exposure and outcome measures. If the exposure–outcome association is consistent and in the expected direction, only 6 twin pairs are needed to detect a significant within-twin pair association (Lachenbruch 2014). Exact McNemar test *P*-value = 0.03 in 6 twin pairs, if for example, the co-twin with amyloid positivity is demented in all pairs in comparison to the amyloid-negative co-twin being non-demented. However, in real data, it is unlikely that the correspondence between exposure and outcome would be perfect and, therefore, larger samples of twin pairs are needed to detect significant associations between exposure and outcome. Furthermore, to make inferences about possible genetic versus environmental confounding both DZ and MZ twin pairs are needed (see Table 1 for different scenarios). Dichotomous measures of exposure and outcome can be determined by using cut-off values for AD pathology (e.g. amyloid-negative versus amyloid-positive) and cognitive status (e.g. dementia versus no dementia). Alternatively, it is possible to assign co-twins within each pair to indicate a co-twin with higher versus a co-twin with lower AD pathology or poorer versus better cognition. The latter approach does not consider if twins are discordant for any predefined threshold, but rather defines the discordance between members of a given twin pair. Although this approach does not match with the established validated cut-offs, it has the benefit of utilizing more twin pairs as it includes also twin pairs with more subtle differences in the analyses (e.g. both members of a twin pair are amyloid-negative but still have different levels of amyloid accumulation). This could, however, result in a scenario where the individual-level associations are not significant, but the within-twin pair associations are significant. For example, in a sample where most twin individuals are non-demented, there may not be an individual-level cortical amyloid–cognition association, but still the within-twin pair associations could be significant (see e.g. Lindgren et al. 2021).

**Table 1:** Within-twin pair associations and genetic and environmental confounding.

	Pattern of associations	Within-twin pair associations
No confounding	IL = within-twin pair	**DZ** = **MZ**
Partial genetic confounding	IL > within-twin pair	**DZ** > **MZ**
Complete genetic confounding	IL > within-twin pair	**DZ** > mz
Partial environmental confounding	IL > within-twin pair	**DZ** = **MZ**
Complete environmental confounding	IL > within-twin pair	dz = mz

Abbreviations: IL, individual-level (between-family effects); DZ, dizygotic; MZ, monozygotic.

Bold font/capital letters indicate statistically significant association in within DZ/MZ pair comparisons. Small letters indicate statistically nonsignificant associations.

Cross-tabulation and the exact McNemar test are the simplest approaches utilizing case–control co-twin design. Using dichotomic outcome, it is also possible to use conditional logistic regression models to investigate if the associations between exposure and outcome are evident within-twin pairs. With adequate sample size, this approach allows to include covariates in the models. Here, the exposure can be either dichotomous or continuous. For example, cortical amyloid pathology as measured with standardized uptake value ratio (SUVR) could be used as a continuous measure in twin pairs who are discordant for clinical AD diagnosis. Conditional regression model corresponds to standard logistic regression model but uses only twin pairs who are discordant for the outcome. Conditional regression models can be used separately in discordant MZ and DZ pairs to see if the within-family associations can be confirmed when controlling for different levels of genetic relatedness. Furthermore, it is also possible to test significant interactions of zygosity, i.e. to test if the within-family association is significantly different in MZ and DZ pairs. Formal testing of similarity/difference in MZ and DZ twin pairs is desired, but in many cases the sample size is too small, especially with regard to MZ pairs. In fact, discordant MZ twin pairs may be hard to find, and very large samples are needed to screen for potential discordant pairs. This is simply because of the fact that AD has a substantial genetic component with heritability as high as 70% (Panizzon et al. 2022), and also brain and cognitive phenotypes are highly heritable (Kremen et al. 2023). To have more statistical power, discordant twin design with continuous outcome can be implemented.

## Continuous outcome

Discordant twin design can also be performed using both exposure and outcome as continuous measures. With continuous measures there is more power, and these models can also include twin pairs who are concordant for either the exposure or the outcome. These models can be implemented with conditional linear fixed effect regression model. Here, it is tested if within-twin pair differences in exposure are related to within-twin pair differences in outcome. Because both variables in the model are continuous also those pairs who are concordant (e.g. having the same score in episodic memory test) can be included. If there exists a within-twin pair association, then it is expected that twin pairs who are concordant for the outcome are also highly concordant for the exposure measure (e.g. highly similar cortical amyloid SUVR). Linear regression models can be implemented in 2 ways, either including or not including the between-family effects in the model (see e.g. Carlin et al. 2005; Frisell et al. 2012 for different models and statistical considerations).

## Interpretation of results from within-twin pair analyses

If the within-twin pair associations are similar to those in individual-level analyses, then there is no confounding and there is more evidence for the causal effect compared with analysis in unrelated individuals. If the within-twin pair associations are attenuated compared with individual-level association then there is evidence for confounding. Genetic confounding occurs when the within MZ pair association is weaker than the within DZ pair association. Complete genetic confounding occurs when the within MZ pair association is not significantly different from 0 (Table 1). Environmental confounding occurs when the within MZ and within DZ associations are both weaker than individual-level association but not significantly different from each other. Complete environmental confounding occurs when both within MZ and within DZ associations are not significantly different from 0 (Table 1).

## Early case reports of twins discordant for AD

For the review, we were interested in studies of twin pairs discordant for either cognition or brain imaging AD biomarker, having both biomarker and cognitive data available. Before discussing the search for our review, we introduce early case studies before the modern era of in vivo MRI and PET imaging.

Discordant twin pair studies in AD research began with case reports dating back to the 1950s; however, these early reports only included autopsies and in some cases electroencephalography (EEG) measures. A case report in 1955 introduced a female MZ twin pair, discordant for AD (Davidson and Robertson 1955). The demented co-twin was diagnosed with probable early onset AD based on her clinical history at the age of 50 years. This twin pair was examined longitudinally using mainly unstructured interviews and clinical examination measures until an autopsy was performed on the demented twin after her passing at 69 years old. The autopsy of the brain showed large, generalized atrophy, particularly in the frontal and parietal lobes, and in cerebellar gray and white matter, and dilation of the ventricles. There were no pathological changes in the small meningeal and cerebral vessels. Fibrillary astrocytes were detected throughout the gray matter, and the cortex showed numerous amyloid plaques. However, there was no information about the brain status of the non-demented co-twin.

**Fig. 1:**
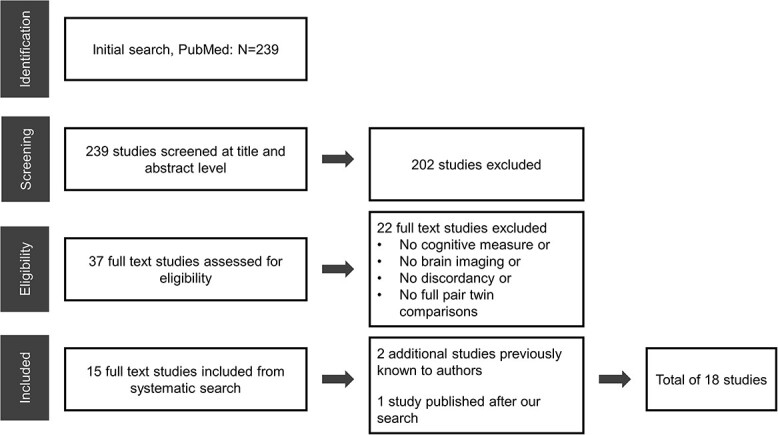
Flow chart of the literature search and included studies.

Another early case report featuring a female MZ twin pair discordant for AD reported similar results (Hunter et al. 1972). An autopsy was performed on the demented twin after her passing at 64 years old, showing significant brain atrophy, most notably in the frontal and occipital lobes. This report used EEG in addition to an autopsy, adding an additional dimension in measurements compared with the first available case report. The co-twin with AD showed severe abnormalities in the background activity during EEG, consisting mainly of theta and delta rhythms with no focal or lateralizing features. The cognitively healthy co-twin demonstrated 11–12 Hz alpha rhythm responsive to visual attention, and the background activity showed some random theta components of similar amplitude. This report used neuropsychological tests such as short story recall and the Wechsler Adult Intelligence Scale to confirm the twins’ cognitive status.

In 1986, another case study featuring a pair of MZ female twins reported similar results (Renvoize et al. 1986). In this case, the proband with AD had early onset dementia at the age of 49 years. The study included EEG and autopsy measures, and a clinical examination that showed impaired short- and long-term memory on the affected twin, whereas her co-twin was cognitively normal. The co-twin with AD showed abnormal activity of theta and delta frequency on EEG, with subclinical seizure activity in 1 recording. At autopsy at the age of 64 years, the affected twin showed cortical thinning with atrophy mostly in the frontal lobes, dilated cerebral ventricles, and presence of cortical amyloid plaques and NFTs. The co-twin remained unaffected until the last follow-up at 73 years old, more than 20 years after the onset of the affected twin's dementia.

## Data availability

As a review article, we did not perform any primary or secondary data analyses. All material used for this review was based on the reported data in published original studies.

## Search method

Our inclusion criteria contained the following: studies included twin pairs discordant for cognition or AD-related brain pathology, used twin-analyses with full pair comparisons, and included cognitive measures and brain imaging for AD biomarkers. We searched for publications on discordant twin pairs in PubMed, with the following search terms: AD, dementia, discordant, twins, co-twin, within-twin, cognitive impairment, cognition, brain imaging, PET, MRI, cortical structure, amyloid, tau, neuroinflammation, and neurodegeneration. The PubMed search was conducted on 2022 April 23. After our literature search, 1 additional study (Coomans et al. 2023) has been published and included in our review that finally covered studies published until 2023 March 9.

The phases of the search can be seen in Fig. 1. First, clearly irrelevant studies were excluded based on title and abstract. Second, the remaining full text studies were assessed independently by AV and discussed with all authors in relation to our inclusion criteria. Additionally, we included 2 studies previously known to the authors and 1 study published after our initial literature search.

We found a total of 18 studies investigating twin pairs who were discordant either for cognition or AD-related imaging markers. A total of 9 studies included participants from the older Finnish Twin Cohort (FTC) study. A summary of all studies can be seen in Table 2. We found twin studies matching our criteria for the following AD imaging biomarkers measured by MRI, PET, and computed tomography (CT): amyloid-β, volume, glucose metabolism, and neuroinflammation (Table 3). Notably, there was only 1 PET study measuring tau pathology in relation to cognition, only 1 structural MRI study looking at CTH, and no studies measuring CSA (Table 3).

**Table 2:** Summary table of discordant twin studies.

**Authors**	**Twin sample**	**Twin cohort**	**Age (years)**	**Cognitive phenotype**	**Brain measures**
Davidson and Robertson (1955)	1 twin pair (MZ) Female	Individual case	Dementia onset at 50	Discordant for AD	Autopsy
Hunter et al. (1972)	1 twin pair (MZ) Female	Individual case	Dementia onset at 49	Discordant for AD	Autopsy, EEG
Renvoize et al. (1986)	1 twin pair (MZ) Female	Individual case	Dementia onset at 49	Discordant for AD	Autopsy, EEG
^a^ Luxenberg et al. (1987)	1 twin pair (MZ) Female	NAS Twin Registry	59 years at study entry	Discordant for AD	CT, FDG-PET, MRI
^a^ Kumar et al. (1991)	3 twin pairs (MZ) 1 female, 2 male	NAS Twin Registry	60, 60, and 61 years at study entry	Discordant for AD	CT, FDG-PET
^a^ Small et al. (1993)	1 twin pair (MZ) Female	Individual case	81	Discordant for AD	MRI, FDG-PET, EEG
^a^ Geroldi et al. (1996)	1 twin pair (MZ)	NA	NA	Discordant for AD	SPET, MRI
^a^ Pedersen et al. (1999)	12 twin pairs (1 MZ, 11 DZ) 9 female, 3 male	Study of Dementia in Swedish Twins	Mean age of dementia onset 69, mean age at the time of scan 75	Discordant for AD	CT
^a^ Lipton et al. (2003)	1 twin pair (MZ), Male	Individual case	67	Discordant for AD	fMRI
^a^ Järvenpää et al. (2003a)	1 twin pair (MZ) Female	FTC	90 years old	Discordant for AD	Amyloid-PET, MRI
^a^ Järvenpää et al. (2003b)	7 twin pairs (MZ) Female	FTC	Mean age 75.1	Discordant for AD	FDG-PET
^a^ Järvenpää et al. (2004)	7 twin pairs (MZ) Female	FTC	Mean age 75	Discordant for cognitive impairment	MRI
^a^ Virta et al. (2008)	9 twin pairs (DZ) 6 female, 3 male	FTC	Mean age 75.9	Discordant for AD	FDG-PET
^a^ Virta et al. (2009)	16 twin pairs (7 MZ, 9 DZ) 13 female, 3 male	FTC	Mean age 75 at study entry	Discordant for AD	FDG-PET
^a^ Scheinin et al. (2011)	17 twin pairs (9 MZ, 8 DZ) 6 female, 11 male	FTC	Mean age 71.6	Discordant for cognitive impairment	MRI, amyloid-PET
^a^ Rossi et al. (2016)	3 twin pairs Female	FTC	68, 76, 76	Discordant for AD	MRI
^a^ Fumagalli et al. (2019)	1 twin pair (MZ) Female	Individual case	Dementia onset at 68, 73	Discordant for dementia age of onset	CT, MRI
^a^ Konijnenberg et al. (2019)	96 twin pairs (MZ, +7 individuals w/o co-twin) 57 female, 42 male	NTR	Mean age 70.5	Cognitively normal	CSF collection, amyloid-PET
^a^ Lindgren et al. (2020)	11 twin pairs (4 MZ, 7 DZ) 6 female, 5 male	FTC	Age range 72–77	Discordant for episodic memory	[11C] PBR28 PET to detect neuro-inflammation
^a^ Lindgren et al. (2021)	45 twin pairs (21 MZ, 24 DZ) 18 female, 27 male	FTC	Mean age 71.6	Discordant for episodic memory	Amyloid-PET
^a^ Coomans et al. (2023)	37 twin pairs (MZ) + 4 individuals w/o co-twin 40 female, 38 male	NTR	Mean age 73.7	Cognitively normal	Amyloid-PET, Tau-PET, MRI

Abbreviations: AD, Alzheimer's disease; CSF, cerebrospinal fluid; CT, computed tomography; DZ, dizygotic; EEG, electroencephalography; FDG, fluorodeoxyglucose; fMRI, functional magnetic resonance imaging; FTC, Finnish Twin Cohort; MRI, magnetic resonance imaging; MZ, monozygotic; NA, not available; NAS, National Academy of Sciences Twin Registry; NTR, Netherlands Twin Registry; PET, positron emission tomography; SPET, single-photon emission tomography.

a Fulfilled the inclusion criteria. Gender and age of participants were not reported for Geroldi et al. because of no access to full text.

**Table 3:** Overview of brain imaging biomarker studies in twin pairs.

	**Twin studies**	**Number of studies**	**Number of twin pairs**
***Amyloid-beta***			
PET	✓	4	147
***Neurofibrillary tangles***			
PET	✓	1	37
***Neurodegeneration***			
MRI			
Volume	✓	7	68
Cortical thickness	✓	1	37
Cortical surface area	✗	NA	NA
CT			
Volume	✓	4	16
PET			
Glucose metabolism	✓	3	20
***Neuroinflammation***			
PET	✓	1	11

Abbreviations: CT, computed tomography; MRI, magnetic resonance imaging; PET, positron emission tomography; NA, not available.

Some studies provide results for multiple brain imaging markers and twin pairs were counted for both markers. Geroldi et al. single-photon emission tomography (SPET) study of glucose metabolism/perfusion counted under PET for glucose metabolism.

## From case reports to studies on discordant twin pairs

The earliest discordant twin pair study involving structural brain imaging and PET imaging for AD biomarkers dates to 1987, when Luxenberg et al. (1987) reported a case of an American, 59-year-old male MZ twin pair discordant for AD. In addition to CT, MRI, and FDG-PET, EEG was also used. Furthermore, they included healthy controls in the study. This report also utilized a variety of cognitive assessments, such as the Mini Mental State Examination (MMSE), Blessed Memory, Information and Concentration Test, Marris Dementia Scale, and Wechsler Adult Intelligence Scale. The demented twin had impairments in all cognitive areas, whereas the unaffected twin showed some impairment only on perceptual organization and delayed recall tests. Moreover, the demented twin showed greater loss of gray matter and enlargement of ventricles compared with healthy controls. The demented twin also had greater glucose hypometabolism in the frontal and parietal lobes and greater metabolic asymmetry compared with both healthy controls and the non-demented twin. The non-demented twin did not differ from controls in regional cerebral glucose metabolism or brain structure.

Another early study utilizing brain imaging on a single pair of MZ twins showed similar findings (Small et al. 1993). An 81-year-old female MZ twin pair discordant for AD was studied. The twins went through initial neuropsychological testing and a 1-year follow-up. Measures for brain pathology included structural MRI, FDG-PET, and EEG. The co-twin with AD showed structural changes in the brain, which were consistent with deep white matter ischemic disease. The FDG-PET showed diminished metabolic rate throughout the brain in the AD twin, with temporal hypometabolism and asymmetry. A third MZ case study of an AD discordant pair reported that both twins showed pathological cortical perfusion as measured with single-photon emission tomography, whereas medial temporal lobe atrophy was seen only in the affected twin (Geroldi et al. 1996). More recently, also Fumagalli et al. (2019) reported a case study with CT and MRI, where both twins showed asymmetric atrophy that was more pronounced in the left hemisphere.

By the end of the twentieth century, twin studies started to feature more participants, and the first study to include more than 1 AD discordant twin pair studied 3 AD discordant twin pairs, adding 2 pairs to the case reported by Luxenberg et al. (1987), and reported similar findings to early individual case studies (Kumar et al. 1991). Next, 12 same-sex twin pairs discordant for AD (11 DZ and 1 MZ pair; 9 female and 3 male pairs) from the Study of Dementia in Swedish Twins were included to investigate structural brain features associated with AD (Pedersen et al. 1999). The mean age of dementia onset in the study was 69 years, and CT scans were performed on average at 75 years. Demented twins were identified using either MMSE or a telephone screening protocol, after which they underwent detailed clinical and neuropsychological testing to confirm their cognitive status. The CT scans revealed greater dilation of temporal horns, lateral ventricles, and the third ventricle in AD twins. The AD twins also showed greater atrophy of the temporal lobes than their healthy co-twins; however, intracranial area or overall brain volume was not associated with the disease status in discordant twin pairs.

## The older FTC study—telephone interviews in screening of cognitively discordant twin pairs from a population-based sample

The above-mentioned Swedish study by Pedersen et al. (1999) utilized telephone interview for cognitive screening. This approach has been used systematically in the older FTC study (Kaprio et al. 2019) that represents about half of the existing co-twin studies of this review. One of the first reports from the FTC study was a case study of a 90-year-old female twin pair discordant for AD (Järvenpää et al. 2003a). Most of the neuropsychological test scores and the regional cerebral glucose metabolic rates of the co-twin with AD were more than 2 standard deviation (SD) units below the mean values of the cognitively healthy reference group, whereas the co-twin without AD did not differ from healthy controls in these measures. The twin with AD also had moderate hippocampal atrophy, whereas the co-twin without AD did not. This twin pair had highly similar life histories, but the co-twin without AD had a history of continuous anti-inflammatory medication (Järvenpää et al. 2003a).

Of course, it is not possible to draw conclusions from a case study of 1 twin pair, but interestingly, subsequent studies in cognitively discordant twin pairs from the older FTC study have confirmed lower glucose metabolism and smaller hippocampal volumes in co-twins with cognitive impairment compared with their co-twins without cognitive impairment (Järvenpää et al. 2004; Virta et al. 2009). Notably, also an association between neuroinflammation and cognition has been found in cognitively discordant twin pairs (Lindgren et al. 2020).

A population-based FTC study has identified cognitively discordant twin pairs by using telephone interview screening of cognitive status by using 2 validated measures, namely telephone assessment for dementia (TELE; Gatz et al. 2002; Järvenpää et al. 2002) and telephone interview for cognitive status (TICS/TICS-m; Brandt et al. 1988; Järvenpää et al. 2002). Discordant twin pairs were then administered in-person neuropsychological test battery and brain imaging including PET imaging with various tracers that capture pathologies implicated in dementia and AD. Twins born before 1938 were screened in 1999–2007 (MEMTWIN I) and twins born in 1938–1944 were screened in 2013–2017 (MEMTWIN II; Lindgren et al. 2021). In MEMTWIN I study, screening was done first in MZ twins and then in DZ pairs resulting in reporting of brain imaging studies including only MZ pairs first. In MEMTWIN II, both MZ and DZ twins were invited from the beginning of the study. Structural MRI and PiB-PET were included for both MEMTWIN I and MEMTWIN II twins, although different scanners were used in these 2 phases of data collections. FDG-PET was included only in twins from MEMTWIN I, whereas PBR-PET was included only in a subsample of MEMTWIN II twins. Both studies included also cognitively healthy non-twin controls.

First reports from the FTC included only MZ pairs. A study including 7 cognitively discordant MZ twin pairs indicated that twins with dementia had about 34% smaller hippocampal volume compared with cognitively healthy controls, whereas their cognitively healthy co-twins had only about 8% smaller hippocampal volume than healthy controls (Järvenpää et al. 2004). Seven demented twins and their non-demented MZ co-twins had also 15 and 10% lower cerebral glucose metabolism in the hippocampus compared with 10 cognitively healthy controls, respectively (Järvenpää et al. 2003b). A study of 3 MZ pairs discordant for late onset AD and 13 non-twin cognitively healthy controls used voxel-wise approach and suggested that in addition to hippocampal volume also volumes in medial temporal lobe, frontal and parietal structures were smaller in probands compared with healthy controls (Rossi et al. 2016). In the unaffected co-twins, frontal and parietal, but not medial temporal, regions were affected (Rossi et al. 2016).

Glucose metabolism has also been found to be significantly lower in the lateral temporal cortex, both in the demented (19%) and non-demented (14%) co-twins from MZ pairs compared with cognitively healthy non-twin controls (Järvenpää et al. 2003b). Subsequent study in 9 same-sex DZ twin pairs indicated that only twins with cognitive impairment but not their cognitively healthy co-twins had lower glucose metabolism compared with cognitively healthy controls (Virta et al. 2008). Cerebral glucose metabolism rate of the demented twins was 20% lower in the hippocampus, 15% lower in the lateral temporal cortex, and 16% lower in the prefrontal cortex compared with cognitively healthy non-twin controls. The non-demented DZ twins showed no such reductions in any cortical region compared with cognitively healthy controls. Finally, a study including both MZ and DZ twin pairs, using voxel-wise analysis, showed that co-twins with dementia as well as their cognitively healthy co-twins had lower glucose metabolism rate compared with healthy controls extensively across the cortex (Virta et al. 2009). Furthermore, cognitively healthy MZ co-twins showed lower glucose metabolism rate than cognitively healthy controls in inferior frontal, lateral temporal, parietal, and medial temporal cortices and, also in the thalamus, putamen, and right amygdala. In contrast, there were no differences between cognitively healthy DZ twins and unrelated cognitively healthy controls (Virta et al. 2009). These differences in cognitively healthy MZ and DZ twins with cognitively impaired co-twins indicate that genetic effects affect glucose metabolism even in the absence of cognitive impairment. Virta et al. (2009) looked also at within-twin pair correlations of glucose metabolism and memory/attention measures in DZ twin pairs. They found statistically significant correlations between differences in co-twins in cognitive tasks and regional cerebral glucose metabolism, namely between hippocampus and delayed memory index (*r* = 0.83, *P* = 0.006) and prefrontal cortex and attention index (*r* = 0.63, *P* = 0.04). No significant correlations were found between lateral temporal and verbal memory (*r* = 0.34, *P* = 0.38), or hippocampus and verbal memory (*r* = 0.50, *P* = 0.18). Correlation analyses between differences in co-twins’ relative regional cerebral glucose metabolism and performance in Wechsler memory scale indexes were only significant for hippocampal relative regional glucose metabolism and delayed memory index (*r* = 0.67, *P* = 0.05).

The first report about cortical amyloid in the FTC included 17 twin pairs (9 MZ and 8 DZ) from MEMTWIN I (Scheinin et al. 2011). The results showed that cognitively preserved MZ co-twins of cognitively impaired co-twins had higher ^11^C-PiB uptake in temporal and parietal cortices and the posterior cingulate compared with 9 cognitively healthy non-twin controls. Cognitively normal DZ twins did not differ from healthy controls. Cognitively preserved MZ twins demonstrated similar ^11^C-PiB uptake patterns as their cognitively affected co-twins, and all cognitively impaired subjects (both MZ and DZ) showed patterns of ^11^C-PiB uptake typical for AD. However, a study including additional participants from MEMTWIN II, with a total of 45 twin pairs and 15 healthy non-twin controls, did not find significant differences between healthy controls and twins (Lindgren et al. 2021).

Two studies in Finnish twins have further looked at twin pairs discordant for episodic memory in relation to PET tracers measuring neuroinflammation (Lindgren et al. 2020) and amyloid pathology (Lindgren et al. 2021). These studies can inform if the associations between clinical and biological characteristics of AD are evident when controlling for shared genetic influences or alternatively implicate that these associations are mediated by genetic effects. In 10 twin pairs, co-twins with poorer episodic memory had about 20% higher cortical neuroinflammation in AD vulnerable regions compared with their co-twins with better episodic memory (Lindgren et al. 2020). A study including 45 twin pairs indicated that within-twin pair differences in cortical amyloid pathology were significantly related to within-twin pair differences in episodic memory: co-twins with poorer episodic memory had greater amyloid-β levels than their co-twins with better episodic memory (Lindgren et al. 2021). Within-twin pairs, those associations were somewhat stronger in DZ than in MZ twins, but there was no statistically significant zygosity-episodic memory interaction. Composite memory score—consisting of 6 episodic memory measures—correlations with cortical amyloid-β load were *r* = −0.49 and *r* = −0.42 in DZ and MZ pairs, respectively (for separate analyses with immediate and delayed recall measures and verbal and visual episodic memory measures see Lindgren et al. 2021). These results suggest that the amyloid–episodic memory association is evident even when controlling for shared genetic effects, thus, supporting the causal role of amyloid-β load on episodic memory impairment and linking biological and cognitive hallmarks of AD. However, larger samples are needed to test for the differences in MZ versus DZ pairs.

## From cognitively discordant twin pairs to healthy twin pairs—insights into preclinical AD

The above reviewed studies have identified cognitively discordant twin pairs, either through clinical diagnosis or by using cognitive screening in population-based samples. Alternatively, it is also possible to select twin pairs who are discordant for brain imaging AD markers. However, this approach may be less cost-effective because very large samples are needed to detect discordant twin pairs. An alternative approach in selecting twin pairs is to purposefully select cognitively healthy pairs and then perform brain imaging. This is a valuable approach for early prediction, as it allows to examine if within-twin pair differences in AD-related biomarkers are related to subtle within-twin pair differences in cognition. This approach has been utilized in the Netherlands Twin Registry (NTR).

## NTR studies of cognitively healthy MZ twin pairs

A discordant twin study that included the highest number of twin pairs so far was conducted through the NTR (Konijnenberg et al. 2019). This study included 96 MZ twin pairs and 7 twins without their co-twin (199 participants). The study investigated the association of amyloid-β PET with memory performance in cognitively normal older adults. Although recruiting of twin pairs was not based on the discordance in cognition, this study was included in our review; first because some of the twin pairs were discordant for cortical amyloid-β aggregation and second, as discussed earlier, it is possible to use continuous measures of brain and cognition to see if within-twin pair differences in these measures are related to each other. All participants of the NTR study were cognitively normal older adults, and the mean age was 70.5 years. The participants were identified as cognitively normal based on several tests for which they had to meet certain cutoff scores. These included TICS-modified score > 23, delayed recall score > −1.5 SD of age- and education-adjusted norms, 15-item Geriatric Depression Scale score < 11, and a Clinical Dementia Rating Score of 0.

Memory performance was assessed with 4 memory tests that have been previously shown to be associated with amyloid pathology. Those tests included the face–name–Associated Memory Examination–names delayed recall (face–name associative memory), Rey complex fig. 3-min recall (visuospatial memory), Rey auditory verbal learning task delayed recall (verbal memory), and the Cambridge Neuropsychological Test Automated Battery Paired Associate Learning (visual associative memory). Cortical amyloid-β load was measured with PET scanning (dynamic [18^F] flutemetamol amyloid-PET scans). These [18^F] flutemetamol images of non-displaceable binding potential (BPND) were categorized as amyloid-negative or positive by visual inspection of 3 radiologists.

Individual-level analysis revealed that poorer performance in the Rey visuospatial memory test was associated with positive amyloid-PET scans (based on visual read), whereas other cognitive tests were not related to cortical amyloid status. The study also included twin analyses using the twin discordance model, with concordant amyloid-negative twin pairs as a control group. There were 14 discordant twin pairs (1 twin with positive amyloid-PET scan and the co-twin was negative), 74 concordant pairs for negative amyloid-PET, and 6 concordant pairs where both twins had positive amyloid-PET scans. Co-twins from amyloid discordant pairs with a negative amyloid-PET scan had a higher BPND compared with twins with a negative amyloid-PET from amyloid-negative concordant pairs. Amyloid-positive co-twins from amyloid discordant pairs showed worse Rey visuospatial memory performance compared with their co-twins who were amyloid-negative (*P* = 0.08).

Twins concordant for an amyloid-positive PET scan had lower Rey visuospatial memory scores compared with amyloid-negative co-twins from amyloid discordant twin pairs (*P* = 0.009) as well as trend level difference with amyloid-negative individuals from amyloid concordant twin pairs (*P* = 0.08). Twins concordant for amyloid-positive PET scans had worse face–name associative memory performance compared with concordant amyloid-negative twins (*P* = 0.02). Twins from discordant pairs who had amyloid-negative PET scans tended to have lower scores on the face–name associative memory test, compared with concordant amyloid-negative twins, but this result was not statistically significant (*P* = 0.07). Twins from concordant and discordant pairs did not show significant differences in paired associative memory, Rey verbal memory performance, and cognitive change index tests. The researchers concluded that face–name associative memory and visuospatial memory could be memory domains sensitive for early AD.

These participants from the NTR were invited to a follow-up study yielding longitudinal amyloid-PET data. Co-twin design study of Coomans et al. (2023) included brain imaging markers from all 3 components of the AT(N) framework: amyloid-β (PET), tau (PET), and neurodegeneration as measured with hippocampal volume and CTH in AD vulnerable regions. Amyloid-PET was available at baseline and at 4-year follow-up allowing to investigate cortical amyloid change. Other analyses were cross-sectional and conducted using the follow-up data that included all imaging markers and cognitive data. These imaging markers allowed for a comprehensive investigation of the AT(N) biomarkers in relation to composite episodic memory score based on 6 memory measures including immediate and delayed recall.

Earlier study in the FTC study had indicated that cross-sectional within-twin pair difference in cortical amyloid are related to within-twin pair differences in episodic memory (Lindgren et al. 2021), and the study of Coomans et al. (2023) replicated this finding in 37 MZ twin pairs (by using composite episodic memory score in line with Lindgren et al., but using a different PET tracer for determining cortical amyloid aggregation). The finding that co-twins with greater cortical amyloid pathology have poorer episodic memory than their co-twins with less cortical amyloid pathology is the first replicated finding in co-twin design studies on the association of biological and clinical hallmarks of AD.

Coomans et al. (2023) have also measured tau and were able to further test if the amyloid–memory association is mediated by tau as suggested by the amyloid cascade hypothesis and the AT(N) framework (Jack et al. 2018). This is what they indeed found: first, within-twin pair differences in cortical amyloid were related to within-twin pair differences in cortical tau and secondly, within-twin pair differences in cortical tau were related to within-twin pair differences in episodic memory and finally, mediation model showed that the within-twin pair differences in tau mediated the association between within-twin pair differences in cortical amyloid and within-twin pair differences in episodic memory. The comprehensive testing of different mediation models (looking simultaneously pathways with tau or hippocampal volume or both tau and hippocampal volume as mediators of amyloid–memory association, and also investigating amyloid as a mediator of tau–memory associations) indicated that the effects of amyloid on memory are mediated by tau (Coomans et al. 2023).

Considering structural MRI measures, Coomans et al. (2023) showed that within-twin pair differences in hippocampal volume were related to within-twin pair differences in episodic memory, thus, extending an earlier finding from the Finnish MZ twins showing that cognitively impaired twins had smaller hippocampal volume than their co-twins with intact cognition (Järvenpää et al. 2004). Within-twin pair differences in cortical amyloid were related to within-twin pair difference in hippocampal volume, but as discussed above, the mediation models indicated that it was tau rather than hippocampal volume that mediated the amyloid–memory association (Coomans et al. 2023).

In addition to tau PET, this study was also the first co-twin study to look at CTH in relation to memory. Co-twins with thinner cortex in AD vulnerable regions of entorhinal, inferior temporal, middle temporal, and fusiform cortices had poorer episodic memory than their co-twins with thicker cortex in these regions (Coomans et al. 2023).

## Conclusions and future perspectives

Despite the long history and availability of twin cohorts, research on brain imaging in cognitively discordant twin pairs in the context of aging and AD-related biomarkers is still in its infancy. We found only 18 studies utilizing the co-twin design when studying the associations of AD-related brain imaging markers and cognitive functioning.

Only few studies in cognitively discordant twin pairs have addressed the common AD imaging markers. MRI findings from the few existing studies have indicated greater hippocampal volume in co-twins without cognitive impairment compared with co-twins with cognitive impairment and there is also evidence that within-twin pair differences in hippocampal volume are related to within-twin pair differences in episodic memory. The association of hippocampal volume with cognition is one of the 2 findings that has been replicated in independent samples using co-twin design. Only 1 twin study so far has looked at CTH (Coomans et al. 2023) and no studies have looked at CSA, a cortical metric that is largely uncorrelated with CTH (Vuoksimaa et al. 2015). As cortical volume is the product of CTH and CSA, both of which follow different trajectories during aging (Vijayakumar et al. 2016), there is a need for more research on these measures alone. In general, CTH has been studied relatively intensively in unrelated individuals, whereas CSA has been less studied. And findings from Coomans et al. (2023) indicated that in line with MRI studies of hippocampal volume, within-twin pair differences in CTH in AD vulnerable regions were related to within-twin pair differences in memory.

The FTC studies that have utilized PET imaging have suggested that lower cortical glucose metabolism rate and higher cortical neuroinflammation and amyloid accumulation are related to poorer episodic memory in within-twin pair comparisons. However, number of MZ and DZ twins in the FTC is small, so these studies have been under-powered to detect significant differences between MZ and DZ twins. Only cognitively healthy MZ pairs have been studied in the NTR, and in line with the findings from the FTC, the results indicated that twins with greater amyloid accumulation had poorer episodic memory than their co-twins with less amyloid accumulation.

Only 2 independent (1 from the FTC and 1 from the NTR) discordant twin studies have looked at cortical amyloid, but these have resulted in replicated finding on the cross-sectional association between cortical amyloid aggregation and episodic memory. Only 1 study (from the NTR) has looked at tau-pathology in relation to cognition, but given the comprehensive assessment including also amyloid-PET and MRI measures of neurodegeneration, this study is to date the most informative in the assessment of causal pathways from biomarkers to cognition in the context of the AT(N) framework. As such, it illustrates the utility and justification for the use of co-twin design in studies of AD-related brain imaging markers and cognition. The comprehensive approach of Coomans et al. (2023) gave further support for the AT(N) framework and suggests that the cascade from amyloid to tau and neurodegeneration to memory impairment is not confounded by shared genetic effects between biomarkers and cognition. Furthermore, that study found that the amyloid–memory association is mediated by cortical tau.

Although the first discordant twin pair studies used EEG measures, this method has been largely neglected for some time in the field of brain imaging of AD. Recently, there have been new projects aiming to use EEG in early detection of AD (Gaubert et al. 2019; Tait et al. 2020; Cejnek et al. 2021). Twin studies could potentially be used in this area of research as well.

Similarly, functional MRI (fMRI) studies in twin samples are rare, but they could be valuable in the context of AD research. We found only 1 report utilizing fMRI in cognitively discordant twin pairs, which examined brain activation patterns in 1 male MZ twin pair during visuospatial and verbal working memory tasks (Lipton et al. 2003). One twin had been diagnosed with AD, whereas his co-twin had remained unaffected. They found greater parietal activation during verbal and visuospatial working memory tasks in the co-twin with AD compared with the cognitively preserved co-twin.

In this review, our focus was on studies that have utilized discordant co-twin design to investigate the associations of brain imaging markers of AD with cognition. However, we are aware that in addition to brain imaging markers of AD, there are other biomarkers beyond the scope of this review, including blood-based biomarkers and CSF markers that can be studied using a co-twin design. For example, a study by Tomassen et al. (2022) investigated CSF levels of amyloid and tau longitudinally in association with cognitive decline in 51 MZ twin pairs and found that amyloid and tau in 1 twin predicted memory decline in the co-twin, and additionally tau markers predicted language decline in the co-twin. Another example of using co-twin design to study the non-imaging biomarkers is a study from Konki et al. (2019) investigating DNA methylation differences in twin pairs discordant for AD. This study found peripheral blood methylation differences in multiple genomic regions between co-twin cases and controls; especially methylation mark in the ADARB2 gene that becomes differentially methylated after disease onset. Finally, co-twin design can be used to investigate brain imaging markers in relation to noncognitive measures to clarify, for example, the associations between risk factors of AD and brain health. Ten Kate et al. (2018) studied 94 MZ twin pairs to investigate the within-twin pair differences in vascular risk factors in relation to within-twin pairs in white matter hyperintensities. Coomans et al. (2022) have also looked at tau PET in relation to dementia risk factors and found that within-twin pair differences in cortical tau (as measured with ^18^F-flortaucipir BPND) were negatively correlated with within-twin pair differences in depressive symptoms, and physical and social activity.

In addition to investigating within-twin pair associations of brain–cognition relationships, co-twin design can be used to compare twins in relation to healthy controls. By including cognitively healthy controls, it is possible to investigate if the co-twins of cognitively impaired twins differ from cognitively healthy controls in brain structure, function, or AD-related pathology. In a scenario whereby healthy co-twins have greater brain atrophy/pathology than healthy controls, there is evidence for the familial effect whereas in case of no differences between cognitively healthy co-twins and healthy controls there is no evidence for the familial effects on brain structure, function, or pathology. Some of the reviewed studies included also cognitively healthy non-twin controls, but the number of participants was small in all studies; thus, not allowing to draw definite conclusions.

Given the existence of multiple well-characterized longitudinal twin studies and cohorts, we propose more systematic utilization of co-twin studies in relation to AD and related dementias to further investigate if the brain–cognition associations are evident when controlling for shared genetic and environmental effects. Specifically, for longitudinal, replication, and novel imaging markers, co-twin studies could enlighten the pathways from brain changes to cognitive changes in the course of AD and help to evaluate the AT(N) (Jack et al. 2018) and the extended ATX(N) (Hampel et al. 2021) framework toward the biological definition of AD.

To conclude, in this review we found only 18 studies using co-twin design that have investigated the associations of AD biomarkers and cognition. The only replicated results in independent samples are the findings that co-twins with smaller hippocampal volume and greater cortical amyloid pathology have poorer memory than their co-twins with greater hippocampal volume and less cortical amyloid pathology (Järvenpää et al. 2004; Lindgren et al. 2021; Coomans et al. 2023). The most convincing evidence (with largest samples and most comparable measures in 2 independent samples) is the relationship between within-twin pair differences in cortical amyloid (measured with PET imaging in both studies) and within-twin pair differences in episodic memory (both studies using a composite memory score including both immediate and delayed recall measures; Lindgren et al. 2021; Coomans et al. 2023); these co-twin design studies support the view that the association of biological (amyloid-β) and clinical marker (episodic memory impairment) of AD is not confounded by genetic or environmental effects. These 2 studies, together with the finding that within-twin pair differences in cortical tau mediate the association between within-twin pair differences in cortical amyloid and within-twin pair differences in episodic memory (Coomans et al. 2023), demonstrate the state-of-the-art of co-twin design in support of the AT(N) framework. Understanding the genetic underpinnings of brain–cognition associations in AD and aging can be valuable in bridging the gap between clinical and biological definitions (A/T(N) framework; Jack et al. 2018) of AD. Case–control co-twin approach would also be a powerful approach in intervention trials to see if treatments, e.g. drugs targeted for amyloid pathology, have effects on cognition when fully controlling genetic predisposition to AD.
